# Lipidomics—Reshaping the Analysis and Perception of Type 2 Diabetes

**DOI:** 10.3390/ijms17111841

**Published:** 2016-11-04

**Authors:** Daniel F. Markgraf, Hadi Al-Hasani, Stefan Lehr

**Affiliations:** 1 Institute for Clinical Diabetology, German Diabetes Center, c/o Auf’m Hennekamp 65, D-40225 Düsseldorf, Germany; daniel.markgraf@ddz.uni-duesseldorf.de; 2 Institute for Clinical Biochemistry and Pathobiochemistry, German Diabetes Center, c/o Auf’m Hennekamp 65, D-40225 Düsseldorf, Germany; hadi.al-hasani@ddz.uni-duesseldorf.de; 3 German Center for Diabetes Research (DZD e.V.), München, D-85764 Neuherberg, Germany

**Keywords:** lipidomics, lipid metabolism, metabolic disorder, diabetes, type 2 diabetes, lipid induced insulin resistance, diacylglycerol (DAG), ceramide (CER)

## Abstract

As a consequence of a sedentary lifestyle as well as changed nutritional behavior, today’s societies are challenged by the rapid propagation of metabolic disorders. A common feature of diseases, such as obesity and type 2 diabetes (T2D), is the dysregulation of lipid metabolism. Our understanding of the mechanisms underlying these diseases is hampered by the complexity of lipid metabolic pathways on a cellular level. Furthermore, overall lipid homeostasis in higher eukaryotic organisms needs to be maintained by a highly regulated interplay between tissues, such as adipose tissue, liver and muscle. Unraveling pathological mechanisms underlying metabolic disorders therefore requires a diversified approach, integrating basic cellular research with clinical research, ultimately relying on the analytical power of mass spectrometry-based techniques. Here, we discuss recent progress in the development of lipidomics approaches to resolve the pathological mechanisms of metabolic diseases and to identify suitable biomarkers for clinical application. Due to its growing impact worldwide, we focus on T2D to highlight the key role of lipidomics in our current understanding of this disease, discuss remaining questions and suggest future strategies to address them.

## 1. Introduction

**Figure 1:**
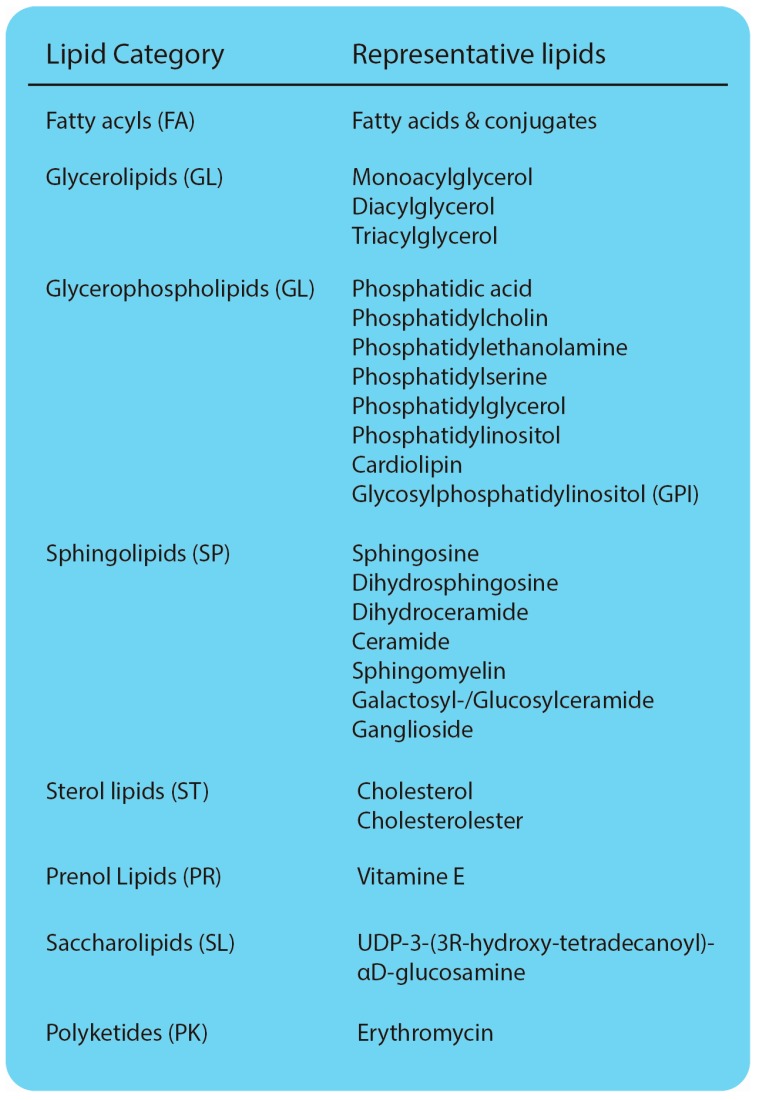
Lipid classes and representative lipids. Classification according to LIPID MAPS^®^ [1].

Lipids present a major group of organic molecules, essential for life. They can be divided into distinct classes and subclasses, highlighting their vast diversity (Figure 1) [1]. The variation of acyl chains and headgroups easily explains the existence of thousands of different lipid species within a eukaryotic cell [2,3]. Lipids fulfill three major functions. First, they serve as energy storage molecules. In particular, the glycerolipid triacylglycerol (TAG) is stored in unique cellular storage organelles, serving as anhydrous energy reservoir [4,5]. Second, lipids are the main constituents of cellular membranes. Glycerophospholipids assemble into lipid bilayers that act as a barrier and ultimately allow the compartmentalization of the eukaryotic cell. Cellular membranes differ with regard to their lipid composition, affecting intrinsic properties, such as fluidity and curvature [6]. Sphingolipids are enriched in special membrane microdomains, further contributing to the local diversification of cellular membranes [7]. Maintaining asymmetry in cellular lipid distribution is fundamental for many processes, ranging from vesicular trafficking to signaling [8]. Finally, lipids act as messenger molecules. The signaling-induced degradation of glycerophospholipids and sphingolipids gives rise to a variety of molecules, such as lysoglycerophospholipids, diacylglycerol (DAG) and ceramides (CER), that are essential transducing components of cellular signaling pathways [9]. Due to their diverse functions, alterations in lipid metabolism can have severe effects on a variety of cellular processes and ultimately cause diseases.

Lipid biosynthesis pathways, i.e., of glycerophospholipids and triacylglycerols, are highly conserved among species. Model organisms, such as yeast, *Saccharomyces cerevisiae*, therefore have been a valuable tool in analyzing cellular lipid metabolism and deciphering the complex and highly regulated interplay between different lipid classes [10,11]. Specific differences in sphingolipid and sterol metabolism, giving rise to different lipid species in yeast and mammalian cells, however, have to be considered when working with model organisms [12,13,14]. Whereas classical work on cellular lipid metabolism was limited to the analysis of defined lipid species or individual classes, rapid progress in mass spectrometry (MS)-based analytics nowadays allows the analysis of the lipidome, the entirety of lipids in a cell [15,16]. Lipidomics, the qualitative and quantitative analysis of the lipidome, therefore provides a powerful tool to answer remaining questions in lipid metabolism and unravel pathological mechanisms underlying metabolic disorders.

## 2. Analytical Approaches to Study Lipids

Due to the high diversity of lipid species, their analysis has been challenging. Initial studies on lipid metabolism were often limited to the analysis of individual lipid classes, using techniques with low sensitivity and resolution, such as thin layer chromatography (TLC). Although still applicable due to low costs and rapid processing, progress in lipid research was driven by the development of MS-based techniques. Within the last decade, many different approaches have been developed and successfully applied to basic and clinical research. Untargeted approaches, covering a large fraction of the lipidome, and targeted approaches, focusing on specific lipid classes and species were successfully applied to discover biomarkers for a variety of diseases, such as cancer, metabolic diseases (discussed below) and cardiovascular disease and to decipher molecular mechanisms underlying lipid metabolism in health and disease. In general, three major lipidomics approaches emerged: (i) direct-infusion lipidomics; (ii) MS coupled with chromatographic separation; and (iii) MS imaging. Here we provide an overview of general procedures and concepts in lipidomics, mainly focusing on direct-infusion- and chromatography-coupled MS techniques relevant for research on metabolic disorders. For further information on MS imaging, we refer the reader to recent reviews [17,18,19].

### 2.1. Preanalytics

**Figure 2:**
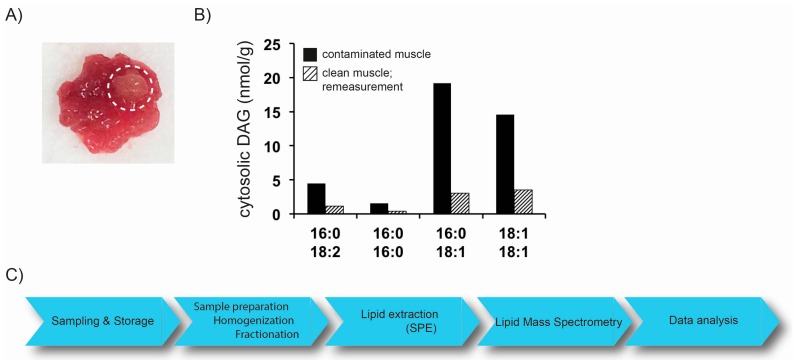
(**A**) Representative image of human muscle (musculus vastus lateralis) biopsy sample. Biopsy was taken using a Bergstrom needle. White circle highlights aggregates of adipocytes; (**B**) analysis of cytosolic DAG concentration using LC-MS/MS. Human muscle biopsy sample with extramyocellular fat contamination was homogenized, fractionated and DAG in the cytosolic fraction were analyzed. A sample from the same biopsy, without any visible extramyocellular fat contamination was processed and analyzed the same way (Interassay variation coefficient of the applied LC-MS/MS method: maximum 4.8%, for the indicated DAG species); (**C**) schematic illustration of lipidomics workflow.

The rapid development of MS-based lipidomics approaches allows the analysis of a plethora of sample materials. The lipidome of cultured cells, covering species from yeast to humans, have been analyzed [16,20]. With the entrance of lipidomics into the field of clinical research, lipidomics techniques were applied to a variety of different tissues and body fluids. Furthermore, the analysis of lipids in subcellular fractions of tissues, such as muscle and liver, plays an important role in research related to T2D (discussed below). Proper preanalytical procedures, covering sample collection, storage, preparation and extraction, therefore have become fundamental, not only for lipidomics analysis. Special care has to be taken when biopsies from tissues, such as muscle, are taken for lipidomics analysis. Sample material has to be carefully inspected for blood vessels and extramyocellular adipocytes. Strict protocols have to be in place to remove such contaminations as they might dramatically influence subsequent quantification of lipids (Figure 2A,B) [21]. Generally, tissue is rinsed with buffers and extramyocellular fat is removed manually. In order to prevent degradation of lipids, sample preprocessing has to occur in a timely fashion and samples should be frozen in liquid nitrogen immediately. Long term storage and repeated freeze/thaw cycles of plasma and serum samples showed little changes in the fatty acid composition of lipid classes, such as cholesterolesters (CE), TAG, and glycerophospholipids (GL) [22,23,24]. However, stability in more complex matrices such as body tissues, might differently affect analyte stability and needs to be considered when conducting studies involving lipidomics approaches. This issue becomes even more important when different omics-approaches are combined in a study. Especially, when dealing with peptides and proteins utilizing proteomic profiling approaches, highly standardized sample processing (e.g., avoiding freeze/thaw cycles or utilizing inhibitors) is crucial for successful analysis. Internal standards (IS), used for quantification of lipid species are added to the sample at the earliest time point possible to account for altered extraction and ionization efficiency. Lipids are then extracted from biological samples to remove interfering compounds, such as saccharides and proteins. Methods developed by Folch et al. and Bligh and Dyer, using chloroform and methanol as extraction solvent, are widely used [25,26]. In addition, two-step extraction and methyl-tert-butyl ether (MTBE) extraction protocols are available [16,27]. Automated lipid extraction systems have been developed and allow the high-throughput analysis of a large number of samples, commonly accumulating in clinical research studies [20]. Refinement of lipid extracts by solid phase extraction (SPE) to separate, e.g., lipid classes prior to analysis, is time-consuming, however, further reduces the risk of ion suppression. Thus, lipidomics analyses comprise a comprehensive set of sequential processes, each tailored for specific applications, that are integrated into an overall methodological workflow (Figure 2C). In order to cover more than one class of molecules in a single workflow, very recently, SIMPLEX (simultaneous metabolite, protein, lipid extraction) was introduced to allow a simultaneous and quantitative analysis of lipids, metabolites and proteins derived from one sample [28]. This strategy, addressing different molecular classes in parallel, paves the way to understand the interaction between lipid metabolism and protein driven signaling processes.

### 2.2. Mass Spectrometry Based Lipidomics

#### 2.2.1. Direct-Infusion MS Techniques

In direct-infusion mass spectrometry, samples, i.e., lipid extracts from biological matrices, are infused into the MS without pre-separation. Described as shotgun lipidomics, this platform was initially developed by Gross and Han, and allows fast and reproducible analysis of lipids in samples [29]. Using a multiplexed lipid extraction, lipid classes can be separated in-source, to enhance performance [30]. Ion suppression caused by those complex sample matrices, especially affects low abundant lipid species and presents a disadvantage of this approach. The advantage of measuring hundreds of lipids in usually limited sample material, however, explains the widespread use of shotgun lipidomics in recent years [16,31,32,33].

#### 2.2.2. MS Coupled with Chromatographic Separation

The chromatographic separation of complex biological samples prior to the MS analysis is widely used to overcome the risks of ion suppression and improve the resolution of isobaric lipid species. Besides TLC, gas chromatography (GC) presents a powerful tool in lipid analytics, still well accepted in fatty acid profiling [34]. Derivatization of lipids, prior to GC analysis, however, might diminish structural information of more complex lipid species. Furthermore, as most lipid species are non-volatile and tend to degrade at high temperatures used in GC, liquid chromatography (LC) systems, coupled to MS detection emerged as powerful tools in lipidomics applications [35,36]. Besides commonly used high performance liquid chromatography (HPLC) systems with normal and reverse phase columns, UHPLC (ultra high performance chromatography), operating at pressures up to 15,000 psi now provide better mass resolution, enhanced sensitivity and greater signal-to-noise ratio [37,38,39]. The chromatographic separation of lipid species reduces ion suppression by reducing the number of competing analytes, entering the MS system at the same time. As retention times for individual lipids are highly reproducible in a given application, methods can be specifically tailored to optimize the analysis time available for individual lipid species. Internal standards for quantification of lipid species have to be selected carefully in order to allow absolute quantification. Due to changing conditions in commonly applied LC gradients, differences in the ionization efficiency of analyte and IS can occur when they elute at different retention times, ultimately affecting quantification results. Finally, carry-over effects on the column can severely affect results, especially when analyzing large numbers of complex biological samples in a row. Methods for the analysis of selected lipid classes and species therefor need to be thoroughly validated in order to account for the possible sources of errors mentioned above.

#### 2.2.3. Identification of Lipid Species by MS

**Figure 3:**
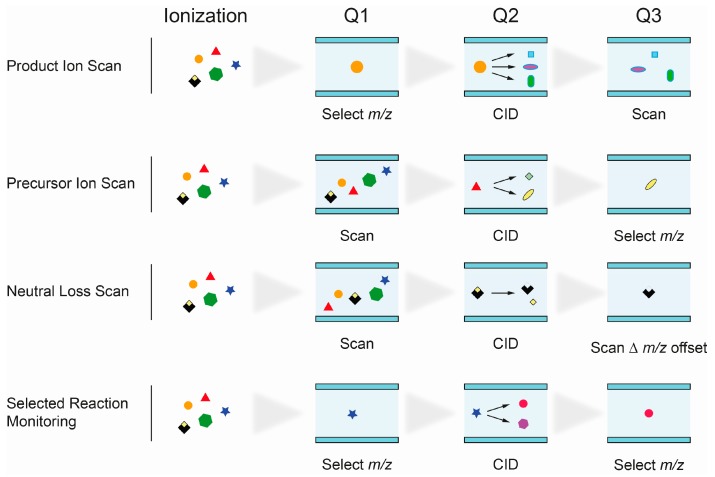
Schematic illustration of scan modes used on triple quadrupole mass spectrometers. Q, Quadrupole; CID, collision induced dissociation.

The unequivocal identification of isobaric lipid species presents a major challenge in lipidomics applications. The use of single mass analyzers, e.g., for the analysis of fatty acids by GC-MS, is therefore not applicable for most, more complex, lipid species. Triple quadrupole (QqQ) mass spectrometers were essential for progress in the field of lipid research and were used for direct-infusion shotgun, as well as for chromatography coupled lipid analysis. The system consists of two mass filters, quadrupoles Q1 and Q2, and a collision cell (Q3). Precursor ions passing the first quadrupole undergo fragmentation in the collision cell (collision induced dissociation, CID) and fragments can be analyzed in the third quadrupole. The linear setup of quadrupoles Q1–3 allows different scan modes for the identification of individual lipid classes and subclasses based on their specific fragmentation behavior (Figure 3).

- Product Ion Scan: fragments of precursor ions, selected in Q1, are analyzed in Q3 after fragmentation in Q2. It is commonly used to study the fragmentation patterns of lipids.
- Precursor Ion Scan (PIS): in this scan mode, precursor ions, which produce a specific, selected fragment ion (Q3), are detected. Since certain lipid classes have common structural motifs, detected as fragment ion in Q3, PIS can be used to distinguish and identify lipid species within them.
- Neutral loss (NL): precursor ions with a specific mass difference between the two mass analyzers Q1 and Q3 are detected. The loss of a fragment, corresponding to a lipid class-specific structural motif, is commonly used to identify lipid species of that particular class.
- Selected reaction monitoring (SRM): SRM is widely used in targeted lipidomics applications because of its high specificity and sensitivity. Selected precursor ions and specific fragment ions are defined for individual lipid species and allow their identification. Multiple reaction monitoring (MRM) is used to analyze multiple lipids. Optimal transitions between precursor and fragment ion are usually determined experimentally, therefore requiring access to reference substances.

Multiple, sequential cycles of fragmentation and product ion analysis of a single precursor ion in MS^n^ experiments can be performed on ion trap mass spectrometry platforms. They provide more detailed structural information on complex lipid species [40,41].

Hybrid quadrupole time-of-flight (QqTOF) mass spectrometers have been used in shotgun lipidomics approaches to overcome limitations of QqQ instruments, which only allow the acquisition of a single PIS or NL per scan. Multiple precursor ion scanning (MPIS) on QqTOF instruments allows the simultaneous acquisition of up to 50 PIS and was applied to characterize the cellular lipidome [20].

The mass spectrometry platforms introduced here, can be combined with a variety of different ionization techniques. Electro spray ionization (ESI) and atmospheric pressure chemical ionization (APCI) present soft ionization technologies that are routinely used and reduce the risk of in-source fragmentation. In summary, the rapid technological development of mass spectrometry-based technologies provides a comprehensive toolkit to establish tailored solutions for the analysis of lipids, a class of highly divers and biologically and clinically relevant metabolites.

## 3. Lipidomics in Metabolic Disease Research

It is now well accepted that many diseases are characterized by dysregulated lipid metabolism. Alterations of lipid profiles can precede the onset of diseases, rather than being a consequence, allowing the development of specific disease biomarkers [42]. Studies utilizing lipidomics to discover biomarkers or unravel underlying cellular pathological mechanisms have increased dramatically over the past years. Lipidomics approaches were applied to study diseases ranging from cancer [43,44], Alzheimer’s disease (AD) [45], cardiovascular diseases [46] to metabolic diseases, such as obesity and T2D [39,47,48]. Herein, we focus on the role of lipidomics in our current understanding of mechanisms underlying T2D, highlight current concepts and discuss future strategies to address remaining questions.

### 3.1. Type 2 Diabetes and Lipid Mediated Insulin Resistance

**Figure 4:**
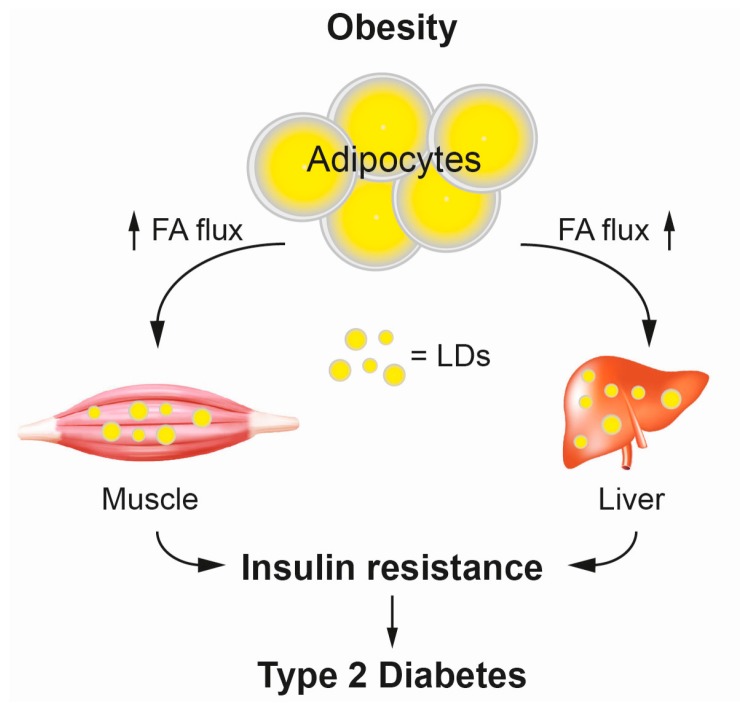
Schematic illustration of the relationship between obesity, ectopic accumulation of fat, insulin resistance and type 2 diabetes. Excessive metabolic energy is stored as TAGs in lipid droplets (LDs) in adipocytes. Extended exposure to high fat environment compromises storage capacity of adipocytes and leads to increased FA flux (upward arrows) and redirection of lipidstowards peripheral tissues, such as muscle and liver. Lipids exceeding the oxidative capacity of ectopic tissue are stored as TAG in LDs. Specific lipids trigger insulin resistance underlying type 2 diabetes. FA, Fatty acids.

**Figure 5:**
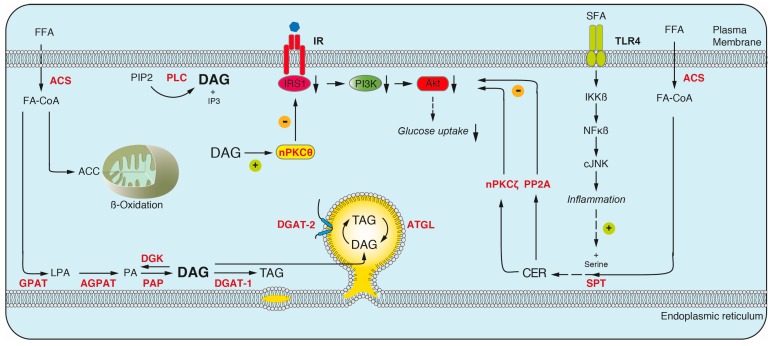
Lipid intermediates and their interactions with insulin signaling pathways in muscle. Free fatty acids (FFA) are transported into the cell, activated by acyl-CoA-synthase (ACS), and channeled into different pathways: (i) TAG synthesis occurs in four sequential reactions, catalyzed by members of the glycerol-3-phosphate-o-acyltransferase (GPAT), 1-acylglycerol-3-phosphate (AGPAT), phosphatidic acid phosphatase (PAP) and diacylglycerolacyl-transferase (DGAT) enzyme families in the ER and/or LDs. DAG, a key intermediate in TAG synthesis, can alternatively be generated by stimulus-dependent cleavage of phosphatidylinositol 4,5-bisphosphate (PIP2) by phospholipase C (PLC) at the plasma membrane. The lipolysis of TAG by adipose triacylglycerol lipase (ATGL) generates DAG on LDs. Accumulated DAG species recruit and activate nPKCθ which leads to inhibitory phosphorylation of IRS1, downregulation of phosphatidylinositol-kinase 3 (PI3K), AKT and ultimately glucose uptake (indicated by downward arrows); (ii) FA-CoAs are converted to acylcarnitines (ACC) for shuttling and subsequent β-oxidation in mitochondria. Decreased or incomplete oxidation leads to their accumulation and affects insulin sensitivity; (iii) Ceramide synthesis is initiated by serine palmitoyl transferase (SPT) in the ER. Binding of saturated FA (SFA) to toll-like receptor 4 (TLR4) induces an inflammatory response, contributing to increased ceramide synthesis. Ceramides inhibit insulin signaling by decreasing the activity of AKT via protein phosphatase 2A (PP2A) or atypical PKCζ. Lysophosphatidic acid (LPA); Phosphatidic acid (PA); Diacylglycerol kinase (DGK); Inositol-1,4,5-Trisphosphate (IP3).

Societies worldwide are facing an obesity pandemic that presents a major burden to public health systems. Obesity is characterized by the excessive accumulation of lipids, i.e., TAGs, in specialized storage organelles, the lipid droplets, in adipocytes and ectopic tissues, such as skeletal muscle and liver. The role of obesity as risk factor for various diseases, such as T2D and cardiovascular disease is undisputed [49,50]. Common to these obesity-associated diseases is the pathogenesis of insulin resistance (IR) [51,52,53]. A role for lipids as mediators of IR was suggested early on [54,55,56]. Studies using lipid infusions in humans reported that lipid infusion increases plasma fatty acid (FA) concentration, intramyocellular lipid accumulation and ultimately causes IR in muscle [57,58]. Interestingly IR associated with defects in glucose uptake but no impairment of glycolysis [59,60,61]. The current working model of lipid-mediated IR suggests, that upon exceeding the maximum storage capacity of adipocytes, lipids are released into the circulation as FAs and transported towards skeletal muscle and liver, where they accumulate in lipid droplets and ultimately trigger insulin resistance underlying T2D (Figure 4) [62,63]. However, the underlying molecular mechanisms linking obesity and IR in T2D are only poorly understood. Interestingly, the ultimate storage lipid TAG appears to be metabolically inert, whereas a variety of other lipid metabolites are implicated in triggering IR in ectopic tissues [64]. Several studies in cell culture models, and animal and human studies have implicated DAGs, CERs, and acylcarnitines (ACCs) as major mediators of lipid-induced IR (Figure 5). As skeletal muscle accounts for up to 90% of insulin-stimulated, postprandial glucose disposal, we here focus on studies utilizing lipidomics approaches to describe pathological mechanisms underlying insulin resistance in muscle.

#### 3.1.1. Diacylglyerol

The glyerolipid DAG is a key metabolite in cellular lipid metabolism. It consists of two fatty acids, esterified with a glycerol backbone, thereby lacking any sizeable headgroup [1]. Different regio- and stereoisomers of DAG exist, with 1,2-DAG being the most abundant species [65,66]. Besides presenting an important intermediate in lipid metabolism, DAGs are functional components of membrane bilayers and serve as signaling molecules. Signaling is mainly mediated by the recruitment and activation of protein kinase C (PKC) isoforms to specific, membrane embedded DAG species [67]. Intracellular DAG levels therefore need to be tightly controlled to balance its various functions. DAG can be synthesized de novo or, alternatively, by cleavage of glycerphospholipids by sphingomyelin synthase or phospholipase C (PLC) [9]. PLC activity is regulated by extracellular stimuli and resulting DAG species are spatially restricted to the plasma membrane [66]. In contrast, de novo DAG synthesis, a key step in the synthesis of TAGs, occurs at the endoplasmic reticulum (ER) by the sequential addition of activated fatty acids, fatty acid-CoenzymeA (FA-CoA), to a glycerol-3-phosphate backbone [68,69]. As the conversion of DAG to TAG can also occur locally on growing lipid droplets emerging from the ER, DAGs are channeled from the ER to newly forming LDs for localized TAG synthesis [70]. Lipolysis of TAG by LD localized lipases, such as adipose triglyceride lipase (ATGL), generates DAG and FAs. DAGs can be further hydrolyzed to provide FAs for energy production in the mitochondria. Alternatively, DAGs are re-esterified to TAG, thereby fueling a futile cycle on LDs between TAG formation and consumption [71,72].

To date, work utilizing a variety of lipidomics approaches established a link between accumulated DAG species and IR in liver and muscle and a general model of DAG mediated, lipid induced insulin resistance emerged. According to the current working model, excess FAs, originating from compromised adipocytes, are taken up by ectopic tissues and are channeled into the TAG synthesis pathway. In muscle, accumulated DAG species were described to recruit novel PKC isoform nPKCθ to the plasma membrane, leading to its activation, subsequent inhibitory phosphorylation of IRS1, thereby inhibiting insulin signaling and ultimately glucose uptake [73,74]. Similarly, in liver, nPKCε was described to be recruited to the plasma membrane in a DAG dependent manner and affect insulin signaling by inhibiting the activation of IRS proteins, the major signal transducing proteins [75].

#### 3.1.2. Ceramides

De novo sphingolipid synthesis is initiated by serine palmitoyl transferase (SPT), which catalyzes the condensation of palmitoyl-CoA and serine in the ER. Subsequent downstream reactions lead to the formation of sphinganine and ultimately ceramide. Alternatively, ceramides can be generated by the hydrolysis of sphingomyelin or the lysosomal degradation of complex sphingolipids in the salvage pathway [76,77]. Besides being important components of cellular membranes, ceramides play an important role as bioactive second messengers [9]. In contrast to the DAG-centered view of lipid induced IR (3.1.1), other studies suggest a role for ceramides in mediating IR [78]. Different mechanisms were described to mediate this process. Cellular ceramide levels can increase upon excessive uptake of FAs and subsequent channeling into the ceramide synthesis pathway. Accumulated ceramide species were shown to reduce AKT activity and thus insulin sensitivity [79]. The effect on insulin sensitivity did not depent on IRS proteins and phosphoinositide 3-kinase (PI3K). Two mechanisms inhibiting AKT were identified. First, the accumulation of ceramides results in the activation of protein phosphatase 2A (PP2A) and ultimately the inhibitory dephosphorylation of AKT [80]. Second, ceramides were shown to prevent the membrane translocation and activation of AKT due to the inhibitory phosphorylation via the atypical PKCζ [79]. Additional studies showed that binding of saturated fatty acids (SFAs) to toll-like receptor 4 (TLR4) triggers the synthesis and accumulation of ceramides via activation of inflammatory pathways, ultimately affecting insulin sensitivity [81,82].

Besides its direct impact on insulin signaling, ceramides contribute to the cell death of insulin producing pancreatic β-cells [83]. Apoptosis, the programmed cell death is initiated by the complex interplay of extrinsic and intrinsic pathways [84]. The binding of death ligands, such as TNF-α, to respective cell surface receptors (e.g., TNF receptor) initiates the extrinsic pathway and was shown to increase ceramide synthesis [85]. Importantly, saturated FAs, which stimulate de novo synthesis of ceramides, were reported to also activate the extrinsic pathway of apoptosis [86]. Ceramides subsequently initiate the intrinsic pathway by increasing the membrane permeability of mitochondria, ultimately leading to the release of apoptogenic factors, such as cytochrome c, and triggering apoptosis via the caspase cascade. Membrane channels that increase its permeability are formed synergistically by ceramides and the proaptototic protein BAX [83,84]. In contrast to the model of membrane channels, formed by BAX oligomers, other studies suggest that ceramides themselves self-assemble in channels that increase membrane permeability [87].

#### 3.1.3. Acylcarnitines

Acylcarnitines are generated by the transfer of activated long chain fatty acids, LCFA-CoA, to carnitine at the mitochondrial membrane. This conversion allows their transport across the mitochondrial membrane by the carnitine shuttle for β-oxidation and subsequent utilization of acetyl-CoA in the TCA cycle. An association between ACCs and IR was shown in human studies [88]. A current model posits that obesity and T2D is accompanied by increased rates of β-oxidation leading to incomplete fat oxidation, impaired switching to carbohydrate oxidation, partial depletion of TCA cycle intermediates, accumulation of ACCs and ultimately IR [89,90]. How ACCs affect insulin sensitivity on a molecular level is currently not known.

Of note, a new class of hydroxy fatty acids (HFAs) with beneficial impact on insulin sensitivity was described recently. Branched fatty acid esters of hydroxyl fatty acids (FAHFA) species, consisting of four FAs and four HFAs in varying combinations, were reported and palmitic acid hydroxy stearic acid (PAHSA) has been positively correlated to insulin sensitivity in adipose tissue and serum of humans [91,92]. The role of PAHSA as biomarker and potential therapeutic strategy to prevent IR and T2D is currently discussed.

#### 3.1.4. The Contribution of DAGs and Ceramides to Insulin Resistance

An overwhelming number of studies analyzed the role of DAGs and CERs in lipid-mediated insulin resistance in animal and human studies. Despite the methodologically, well-described impact of each lipid class on insulin sensitivity in individual studies, seemingly contradicting results exist between studies. Increased levels of DAGs and CERs were reported in obese insulin resistant, compared to healthy lean subjects [93]. However, muscle ceramides Cer(d18:1/18:0) were increased in insulin resistance humans, independent of obesity [39]. In addition to Cer(d18:1/18:0), Cer(d18:1/16:0) was increased in muscle of obese women, indicating gender-specific differences [94]. Studies in mice support the chain-length specific effects of Cer(d18:1/18:0) and Cer(d18:1/16:0) on glucose tolerance [95,96,97]. In contrast, no changes in CER concentration in muscle of insulin resistant individuals were observed in other studies [98,99,100]. Several studies, addressing the subcellular dynamics of lipids consistently reported a role for DAGs in lipid mediated insulin resistance in humans. Bergman et al. reported increased membrane DAGs, DG(18:0 20:4), DG(16:0 16:0), DG(18:0 18:0), in T2D subjects, which positively correlated with IR [101]. The conclusion that saturated DAG species within muscle correlate with insulin resistance was unexpected, as DAG species with at least one unsaturated FA (UFA) are better activators of PKC [102]. However, consistent with the dissociation of IR from the classical nPKCθ pathway in this study, no correlation between DAG concentration and nPKCθ activation was found. An increase in cytosolic and membrane DAGs in T2D humans was described by Szendroedi et al. [103]. Interestingly, membrane DAGs, DG(18:0 20:4), DG(18:0 18:2), DG(18:1 18:2), and DG(18:2 18:2), as well as 16:0 and 18:2 containing DAGs correlated with IR, even after adjustment for BMI. Strikingly, a strong relationship between UFA containing DAG species (C18:2, C20:4-containing) and nPKCθ activation was described [103]. These results were in line with results in skeletal muscle of lipid infused healthy humans. Membrane DAG, DG(18:2 18:2), and total C18:1-, C18:2-, and C18:3-containing DAG species accumulated in human muscle after 5 and 6 h lipid infusion, respectively [104,105]. Serial muscle biopsies during lipid infusion lead to an increase in membrane and cytosolic DAG, DG(18:1 18:1), DG(18:1 18:2), DG(18:2 18:2), DG(16:0 18:2), and DG(18:1 16:0) at 2.5 h, while levels of ceramides and acylcarnitines remained unchanged. Interestingly, nPKCθ activation and inhibitory IRS1 phosphorylation increased at 4 h. Concomitantly, the activation of major insulin signaling transducing molecules, i.e., the phosphorylation of PI3K and AKT, decreased. This study currently provides the most time-resolved picture of the events underlying lipid induced insulin resistance. Future studies need to address the spatiotemporal dynamics of IR-relevant lipids and their effector proteins in more detail. Currently applied subcellular fractionation techniques are not sufficient to distinguish between subcellular membranes, but rather separate crude membrane mixtures (e.g., ER, mitochondria and plasma membrane) and cytosol. Accordingly, the accumulation of DAG and recruitment of nPKC isoforms specifically to the plasma membrane, as suggested in current working models, has not yet been shown. Considering that TAG synthesis occurs at the ER and LDs, it is rather likely that the intermediate metabolite DAG accumulates at this subcellular localization. How the recruitment of nPKC isoforms to DAG in the ER and LDs affect insulin signaling processes initiated at the plasma membrane is a fascinating question. Lipidomics, in combination with proteomic tools and advanced subcellular fractionation techniques to separate LDs, cytosol and organelle membranes will be essential to address the spatiotemporal dynamics of DAGs and its effector proteins underlying lipid induced IR in future studies.

## 4. Lipidomics, One Member of the -Omics Family

The targeted analysis of lipids in human tissue samples is essential for understanding the pathological mechanisms underlying diseases. In this context the identification and development of biomarkers will help to assess individual risk factors and predict disease progression in the future. Recent lipidomics analysis of human blood of well phenotyped subjects identified lipid signatures predicting ectopic fat deposition and insulin resistance (Table 1). As shown in Table 1, the unequivocal identification of clinically relevant biomarkers is hindered by seemingly inconsistent results between studies, which, most likely, result from different study designs. In particular the effects of gender, age and ethnicity on the lipidome are well documented in the literature and have to be carefully considered when planning, conducting and comparing studies [106,107,108]. To gain deeper insight into pathophysiology and the underlying molecular mechanisms, the parallel analysis of different molecule species, i.e., lipids, proteins, nucleic acids and metabolites, is indispensable. Accordingly, integrative multi-omics approaches will uncover changes within the complex interaction network, built by the different molecule species at a system biology level.

Substantial improvements in mass spectrometry instrumentation and bioinformatic workflows allow performing comprehensive and quantitative analysis of lipids, proteins and metabolites from an identical sample. For example, with the very recent introduction of a novel lipid screening platform (Lipidyzer^TM^, Sciex) [109], accurate parallel quantification of over thousand lipids, utilizing MRM, is now available. The system workflow comprises all steps of the analysis, i.e., beginning with standard operating procedures (SOP)’s for sample preparation up to dedicated software solutions and internal standards.

Complementary, corresponding protein profiles can be dissected by a novel targeted mass spectrometry technique, called data independent acquisition (DIA-MS) or SWATH-MS^®^ [110]. This approach utilizes the information of fragment ion spectral libraries (generated by data dependent MS methods) to mine the complete fragment ion maps acquired by using data independent acquisition method. Accuracy and consistency of quantification is comparable to that of selected reaction monitoring, which presents the gold standard method for proteomic quantification. Moreover, the technology allows a dynamic extension of the acquired data set and re-quantification over time without losing reliability. Last but not least Absolute IDQ^®^ (Biocrates) assays provide a complete workflow for quantification up to 188 endogenous metabolites including different compound classes (i.e., ACCs, amino acids, hexoses, glycerophospholipids and sphingolipids and biogenic amines). Combining such MS-based approaches will contribute significantly to our understanding of disease pathophysiology and open up the opportunity to identify lipid, protein and metabolite biomarkers in plasma. Accordingly, this may represent the first step on the way to monitor individual’s health status from a single drop of blood.

## Figures and Tables

**Table 1:** Lipid biomarker discovery in T2D and obesity.

Reference	Volunteer	Sample	Increase	Decrease
[48]	OIR vs. OIS	Serum	LacCer(22:0) SPM(18:1) SPM(24:1)	–
[39]	Obese vs. lean (m/f)	Plasma	dCer(d18:0/22:0) TAG(16:0/18:1/18:1) DAG(18:0/20:4)	PC(32:0) PC(34:2) PC(36:2) lyso-alkyl-PC(24:2) lysoPE(16:0) acyl-alkyl-PC
	OIR vs. OIS (m/f)	Plasma	DAG(14:1/16:0) CE(22:4) lyso-alkyl-PC(35:4)	Hex2-Cer(d18:1/22:0) Hex2-Cer(d18:1/24:0) lysoPC(22:0)
[47]	T2D vs. Ctr (m/f)	Plasma	CE(23:2) CE(23:3) CE(23:4)	PE(36:4) PE(36:5) PE(36:6)
[111]	T2D vs. NGT	Plasma	dCer, Cer, PE, PI, PG, CE, DAG, TAG	acyl-alkyl-PC
	Prediabetes vs. NGT	Plasma	dCer, Cer, PE, PI, PG, CE, DAG, TAG, free cholesterol	acyl-alkyl-PC
[112]	T2D prospective study, maximum 23.35 year follow-up (m/f)	Plasma	dCer(d18:0/18:0) lysoalkyl-PC(22:1) TAG(16:0/18:0/18:1)	–
[113]	T2D prospective study, 7 year follow-up (m/f)	Serum	PC(32:1) PC(36:1) PC(38:3) PC(40:5)	SPM(16:1) lysoPC(18:2) acyl-alkyl-PC (34:3; 40:6; 42:5; 44:4; 44:5)
[42]	T2D < 1 year diagnosis vs. Ctr (m/f)	Plasma	FFAs: (18:1w9, 18:4w3, 20:4w6, 22:4w6) ACC(C3) ACC(C4) ACC(18:2)	FFAs: (10:0; 13:0,; 14:1w5) SPM(16:1) SPM(OH)(14:1) PC(38:3) PC(44:3) PC(42:1) PC(42:2) lysoPC(28:1)
[114]	T2D prospective study, 12 year follow-up (m/f)	Plasma	TAG (low carbon & double bond number)	TAG (high carbon & double bond number)

OIR, Obese insulin resistant; OIS, Obese insulin sensitive; LacCer, Lactosylceramide; SPM, Sphingomyelin; Cer, Ceramide; dCer, Dihydroceramide; PC, Phosphatidylcholine; PE, Phosphatidylethanolamine; CE, Cholesterolester; Hex2-Cer Dihexosylceramide; FFA, free fatty acid; AAC, Acylcarnitine; PI, Phosphatidylinositol; PG, Phosphatidylglycerol; NGT, Normal glucose tolerant, m, male; f, female; Ctr, control.

## Abbreviations

ACC	Acylcarnitine
ACS	Acyl-CoA synthase
AD	Alzheimer’s disease
AGPAT	1-Acylglycerol-3-phosphate acyltransferase
AKT	Protein kinase B (PKB)
APCI	Atmospheric pressure chemical ionization
ATGLCE	Adipose triglyceride lipase Cholesterolester
CER	Ceramide
CID	Collision induced dissociation
CoA	Coenzyme-A
DAG	Diacylglycerol
DGAT	Diacylglycerol acyltransferase
DGK	Diacylglycerol kinase
ESI	Electrospray ionization
ER	Endoplasmic reticulum
FA	Fatty acid
FAHFA	Fatty acid hydroxyl fatty acid
GC	Gas chromatography
GL	Glycerophospholipids
GPAT	Glycerol-3-phosphate-o-acyltransferase
HFA	Hydroxy fatty acid
HPLC	High performance liquid chromatography
IP3	Inositol-1,4,5-Trisphosphate
IR	Insulin resistance
IRS	Insulin receptor substrate
LC	Liquid chromatography
LCFA	Long chain fatty acid
LD	Lipid droplet
LPA	Lysophosphatidic acid
MTBE	Methyl-tert-butyl ether
MPIS	Multiple precursor ion scanning
MRM	Multiple reaction monitoring
MS	Mass spectrometry
NL	Neutral loss
PA	Phosphatidic acid
PAHSA	Palmitic acid hydroxyl stearic acid
PAP	Phosphatidic acid phosphatase
PIS	Precursor ion scanning
PIP2	Phosphatidylinositol 4,5-bisphosphate
PI3K	Phosphoinositide-3 kinase
PKC	Protein kinase C
PLC	Phospholipase C
PP2AQqQ	Protein phosphatase 2 ATriplequadrupole
SCFA	Short chain fatty acid
SFA	Saturated fatty acid
SP	Sphingolipid
SPT	Serine palmitoyl transferase
ST	Sterol lipids
TAG	Triacylglycerol
TLC	Thin layer chromatography
TLR4	Toll-like receptor 4
TOF	Time-of-flight
T2D	Type 2 diabetes
SIMPLEX	Simultaneous metabolite, protein, lipid extraction
SPE	Solid phase extraction
SRM	Selected reaction monitoring
TNF-α	Tumor necrosis factor α
UFA	Unsaturated fatty acid
UHPLC	Ultra high performance liquid chromatography
